# Prognostic Analysis and Influencing Serum Biomarkers of Patients With Resectable Pancreatic Adenosquamous Cancer

**DOI:** 10.3389/fonc.2020.611809

**Published:** 2021-01-15

**Authors:** Yusheng Shi, Xinjing Wang, Weize Wu, Junjie Xie, Jiabin Jin, Chenghong Peng, Xiaxing Deng, Hao Chen, Baiyong Shen

**Affiliations:** ^1^Department of General Surgery, Pancreatic Disease Center, Ruijin Hospital, Shanghai Jiaotong University School of Medicine, Shanghai, China; ^2^Research Institute of Pancreatic Diseases, Shanghai Jiaotong University School of Medicine, Shanghai, China; ^3^State Key Laboratory of Oncogenes and Related Genes, Shanghai, China; ^4^Institute of Translational Medicine, Shanghai Jiaotong University, Shanghai, China

**Keywords:** Longterm survival, pancreatic surgery, Pancreatic Cancer, overall survival, Pancreatic adenosquamous cancer

## Abstract

**Objectives:**

There are few reports about the survival rate of patients with pancreatic adenosquamous cancer (PASC). This study evaluated and analyzed prognostic factors of patients with resectable pancreatic adenosquamous cancer (rPASC), which might fulfill the blank in the research of PASC.

**Methods:**

In this study, we identified and analyzed 55 patients who were diagnosed with rPASC from January 2013 to May 2019 at the Pancreatic Disease Center of the Shanghai Ruijin Hospital affiliated with Shanghai Jiaotong University School of Medicine. Age, sex, BMI, tumor position, and other important demographic data were collected and analyzed. The follow-up was updated by December 31th, 2019 with a median follow-up of nine months.

**Results:**

Among the 55 patients, 23 (41.8%) patients were female, and the mean age was 62.0 ± 10.3 years. The median overall survival (OS) time was 10 ± 2.1 months, and the median disease-free survival (DFS) time was 4 ± 0.9 months. The 1-year, 3-year, and 5-year survival rates were 40.9, 17.5, and 11.6%, respectively. The multivariate analysis showed that normal serum level of Ca199 (HR = 0.464, 95% CI = 0.222–0.970, P = 0.041) and Ca125 (HR = 0.441, 95% CI = 0.233–0.835, P = 0.012) were independent favorable prognostic factors.

**Conclusion:**

Patients with rPASC had poor survival. The 5-year survival rate was only 11.6%. Normal serum levels of Ca199 and Ca125 were independent favorable prognostic factors that predicted prognosis.

## Introduction

Pancreatic adenosquamous carcinoma (PASC) is a rare type of malignant primary epithelial carcinoma of the pancreas (1). The incidence of PASC only accounts for about 1–4% of pancreatic cancer (2). The pathological manifestation is that a number of squamous cells are mixed with the adenocarcinoma cell population (3). The diagnostic criteria is that squamous cells account for at least 30% of all tumor cells in the carcinoma (1). PASC usually originates from glandular epithelium such as stomach, colon (4). We reported the genomic signatures of PASC and showed that PASC carried highly enriched TP53 mutations and 3p loss compared to PDAC in 2017 (5).

Like pancreatic adenocarcinoma (PDAC), there are no typical symptoms at an early stage which makes it hard to diagnose (6). Some patients suffer from jaundice, abdominal pain, nausea, and other digestive symptoms. CT and MRI are important non-invasive method to verify the tumor. The tumor of PASC is usually more aggressive than that of PDAC (2, 7). Now endoscopic ultrasound (EUS) combined with fine-needle aspiration (EUS-FNA) is often provided for suspected patients however it is not 100% accurate (8, 9).

Based on previous reports, the prognosis of PASC is worse than that of PDAC (2, 6, 7, 10) with a 5-year survival rate less than 8%. In 2012, a population-based study was firstly reported to evaluate outcomes in PASC (2). They revealed that surgical resection was the most important factor which related to the long-term survival. In a recent study from the SEER database, the chemoradiotherapy was associated with better prognosis in patients with resected PASC (11). In patients who received chemoradiotherapy, the median survival time was 23 months. However, the authors also admitted the limitation of their results. In another study of only 25 patients with PASC, the median overall survival was only 8.2 months (12). There have been few studies with a large sample size on long-term outcomes of patients with resectable PASC (rPASC) or serum biomarkers related to the prognosis of rPASC patients to date.

## Materials and Methods

### Patient Selection and Study Design

In total, 62 patients were diagnosed with rPASC by radiological exams from January 2013 to May 2019 at the Pancreatic Disease Center of the Shanghai Ruijin Hospital affiliated with Shanghai Jiaotong University School of Medicine. For rPASC, we did not administer neoadjuvant chemotherapy to patients before surgery as first-line therapy. Among the total cohort, five patients were excluded because of distant metastasis upon surgical exploration, and two patients were excluded due to a loss of follow-up. A total of 55 patients were included, and all of them received R0 resection.

Age, sex, body mass index (BMI), tumor position, and other important demographic data were collected and analyzed. Preoperative levels of hemoglobin and albumin were also included because they revealed patients’ nutriture. Positive symptoms referred to symptoms such as abdominal pain, nausea, vomiting, or any other type of discomfort that would lead the patient to visit doctors. Levels of tumor biomarkers were divided according to the diagnostic criteria in China. Data were stratified according to the diagnostic criteria or former reports to perform survival analysis. The demographic data are displayed in **Table 1**.

**Table 1:** Demographic data and univariate analysis of risk factors.

Variable	n	Mean Overall Survival m, (sd)	95% CI	P value	Median Overall Survival m, (sd)	Mean Disease-Free Survival m, (sd)	95% CI	P value	Median Disease-Free Survival m, (sd)
Age									
<60	23	19.1 (4.8)	9.7–28.4	0.883	11 (2.5)	11.1 (4.3)	2.7–19.6	0.886	6 (2.9)
≥60	32	19.2 (4.6)	10.3–28.2		9 (3.4)	14.6 (4.6)	5.5–23.7		4 (0.7)
Sex									
Male	33	20.0 (4.8)	10.6–29.4	0.795	11 (3.6)	14.4 (4.6)	5.3–23.5	0.696	4 (1.4)
Female	22	17.0 (3.4)	10.3–23.6		9 (2.4)	10.7 (3.3)	4.2–17.2		5 (1.5)
BMI									
<19	8	19.8 (5.9)	8.2–31.5		10 (8.2)	11.4 (3.3)	4.9–17.9		6 (8.0)
19–24	34	18.4 (3.4)	11.8–24.9	0.766	11 (2.1)	10.9 (3.0)	5.1–16.8	0.543	4 (1.0)
≥24	13	22.8 (9.8)	3.6–42.0		9 (1.9)	14.8 (9.4)	0–33.1		4 (1.2)
Symptom									
Positive	39	14.9 (2.3)	10.3–19.5	0.442	9 (3.4)	8.2 (2.0)	4.2–12.1	0.164	4 (1.0)
Negative	16	16.3 (7.9)	10.8–41.8		10 (1.8)	24.9 (8.4)	8.4–41.4		5 (2.0)
Position									
Head	30	25.4 (6.5)	12.7–38.2	0.967	6 (1.7)	17.0 (5.7)	5.8–28.2	0.956	4 (0.9)
Body/Tail	25	17.0 (3.5)	10.2–23.8		11 (1.5)	12.2 (3.8)	4.6–19.6		6 (0.8)
Hemoglobin									
Normal	27	19.4 (3.8)	12–26.9	0.460	12 (3.0)	13.1 (3.7)	5.8–23.3	0.937	5 (1.7)
Abnormal	28	22.6 (6.3)	10.2–35.0		6 (2.0)	13.6 (4.0)	5.7–21.5		4 (0.9)
Albumin									
Normal	44	19.1 (3.1)	13.0–25.2	0.322	12 (2.2)	12.1 (2.9)	6.5–17.8	0.673	5 (1.3)
Abnormal	11	20.2 (9.1)	2.3–38.0		6 (1.2)	17.5 (9.5)	0–36.1		4 (0.7)
DM History									
Yes	28	24.5 (6.8)	11.3–37.8	0.307	13 (3.0)	12.9 (5.3)	2.6–23.3	0.937	5 (1.7)
No	27	16.3 (3.7)	9.0–23.6		9 (3.1)	13.6 (4.0)	5.7–21.5		4 (0.9)
Serum Amylase									
Normal	41	19.6 (4.4)	11.0–28.2	0.510	9 (2.3)	15.1 (4.5)	6.3–23.9	0.579	4 (1.0)
Abnormal	14	17.7 (4.1)	9.7–25.7		12 (4.7)	9.7 (2.7)	4.4–14.9		4 (3.3)
Ca125 Level									
<35 U/ml	31	28.8 (6.1)	16.8–40.8	0.008	18 (5.5)	22.2 (6.0)	10.3–34.0	0.010	5 (2.2)
≥35 U/ml	24	9.4 (1.5)	6.5–12.3		9 (1.6)	3.9 (0.6)	2.7–5.0		4 (1.2)
Ca199 Level									
<35 U/ml	13	27.7 (6.9)	14.2–41.2	0.043	24 (5.8)	21.2 (6.9)	7.7–34.7	0.042	8 (1.7)
≥35 U/ml	42	17.4 (4.0)	9.5–25.2		7 (1.7)	11.3 (3.7)	4.1–18.6		3 (0.7)
CEA Level									
<5 ng/ml	38	26.8 (5.7)	15.6–38.0	0.121	11 (1.9)	19.7 (5.4)	9.1–30.3	0.085	5 (1.8)
≥5 ng/ml	17	12.4 (3.3)	5.8–18.9		6 (4.5)	6.3 (2.9)	0.7–11.9		4 (1.4)
Smoking History									
Yes	13	8.5 (1.5)	5.6–11.5	0.022	9 (4.1)	3.2 (0.8)	1.6–4.9	0.019	2 (0.9)
No	42	25.3 (5.2)	15.1–35.5		11 (3.4)	18.0 (4.8)	8.7–27.4		5 (0.9)
Drinking History									
Yes	10	11.2 (2.7)	4.9–16.5	0.626	6 (4.1)	4.8 (1.3)	2.3–7.3	0.341	2 (0.8)
No	45	21.8 (4.5)	13.1–30.5		10 (2.2)	17.0 (4.5)	8.1–25.8		5 (0.8)
Tumor size									
<4cm	24	22.5 (5.9)	10.9–34.2	0.616	9 (1.4)	15.1 (5.4)	4.6–25.7	0.679	5 (1.2)
≥4cm	31	16.6 (3.8)	9.2–24.0		11 (3.1)	11.9 (3.8)	4.5–19.4		4 (1.4)
Lymph nodes									
Negative	30	20.8 (4.9)	11.2–30.2	0.438	12 (1.9)	15.0 (4.9)	5.5–24.6	0.514	5 (0.8)
Positive	25	18.4 (4.8)	9.0–27.8		6 (0.5)	12.2 (4.4)	3.7–20.8		3 (0.6)
Chemotherapy									
Yes	30	28.9 (6.5)	16.2–41.6	0.041	14.0 (3.2)	18.0 (5.7)	6.8–29.1	0.601	5 (1.4)
No	25	13.4 (2.5)	6.6–20.3		6 (1.5)	11.4 (3.8)	3.9–18.8		4 (0.8)

All of these patients were interviewed by telephone or in the outpatient department every 3 months and provided thorough information. Regular blood tests, liver function tests, renal function tests, tumor biomarker measurements, including Ca199, Ca125, CEA, or AFP, and enhanced computed tomography (CT) or MRI were routinely performed. Exams of non-local patients were performed at local hospitals, and the results were sent to the researchers. Specific new-onset tumors or metastases detected by CT, MRI, or positron-emission tomography-CT (PET-CT)/MRI were considered recurrences. When patients suffered from abdominal pain, jaundice, or other symptoms at any time, they were asked to immediately undergo blood tests and radiological tests. The follow-up was completed by December 31, 2019. The median follow-up was 9.0 (4.0–84.0) months.

This study was approved by the institutional review board of Shanghai Ruijin Hospital. The informed consent was signed by every patient as the agreement for taking the operation and the use of the data we collected before and after surgery.

### Statistical Analysis

SPSS™ 23.0 (IBM, Chicago, IL, USA) was used for all calculations. GraphPad PRISM™ (GraphPad Software, San Diego, CA, USA) was used for plotting. Survival data were analyzed using the Kaplan-Meier method, and curves were compared using the log rank test. Multivariate survival analyses were performed using a Cox proportional hazards model. P < 0.05 was considered statistically significant.

## Results

### Demographic Data and Pathology

Among the 55 patients, 23 (41.8%) patients were female and the mean age was 62.0 ± 10.3 years. Thirty-nine (70.9%) patients suffered digestive symptoms before diagnosis, which primarily included abdominal or back pain. Thirteen (23.6%) patients had a smoking history, and 10 (18.2%) patients had a drinking history. Elevated Ca199 was observed in 42 (76.4%) patients, while elevated Ca125 occurred in 24 (43.6%) patients. Tumors located at the pancreatic head were found in 30 (54.5%) patients, and 25 (45.5%) patients had tumors in the pancreatic body and tail. The mean tumor size was 4.5 ± 2.2 cm. Tumor stages T1, T2, T3, and T4 were found in 6 (10.9%), 25 (45.5%), 21 (38.2%), and 3 (5.5%) patients, respectively. Stages N0, N1, and N2 were found in 31 (56.4%), 18 (32.7%), and 6 (10.9%) patients, respectively. Twenty-eight (50.9%) patients had a diabetes mellitus (DM) history, and serum amylase was abnormal in 14 (25.5%) patients.

### Morbidity, Chemotherapy, and Survival

Eight patients suffered from grade B pancreatic fistula, while two patients suffered from biochemical leakage after surgery. Bile leakage occurred in two patients. Abdominal infection occurred in 13 patients and no delayed gastric emptying (DGE) happened. All the patients recovered, and no perioperative death occurred. Thirty patients received chemotherapy with regimens of Abraxane+gemcitabine (AG), gemcitabine+S-1 (GS), gemcitabine alone, or S-1 alone. Radiotherapy was not provided in this study. The median overall survival (OS) time was 10 ± 2.1 months, and the median disease-free survival (DFS) time was 4 ± 0.9 months. The 1-year, 3-year, and 5-year survival rates were 40.9, 17.5, and 11.6%, respectively. Only four patients survived for more than 3 years, and two patients survived for more than 5 years.

### Prognostic Factors of Overall and Disease-Free Survival

In the univariate analysis of OS time, the normal Ca199 level (p = 0.043), normal Ca125 level (p = 0.008), no-smoking history (p = 0.022), and receiving chemotherapy (p = 0.041) were favorable prognostic factors. The p-value of the CEA level was slightly higher than 0.05 (p = 0.121). Multivariate analysis showed that Ca199 <35 U/ml [hazard ratio (HR) = 0.387, 95% confidence interval (CI) = 0.168–0.890, P=0.025] and Ca125 <35 U/ml (HR = 0.374, 95% CI = 0.187–0.749, P = 0.005) were independent favorable prognostic factors. The results are displayed in **Table 2**.

**Table 2:** Multiple analysis of PFS in PASC patients.

	Hazard ratio	95% CI	P value
Ca125 <35 U/ml	0.441	0.233–0.835	0.012
Ca199 <35 U/ml	0.464	0.222–0.970	0.041
Smoking history	1.391	0.656–2.949	0.390
CEA <5 ng/ml	0.758	0.399–1.440	0.397
Symptomatic	0.839	0.398–1.769	0.644

**Figure 1:**
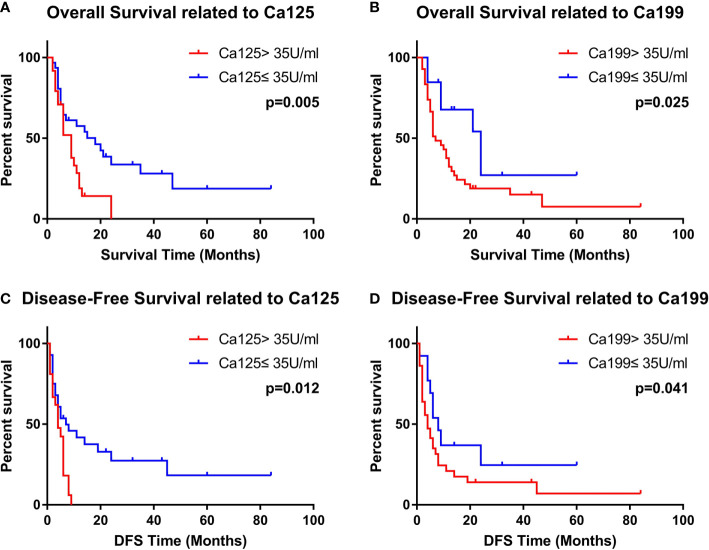
The Relationship between serum level of Ca199 and Ca125 and the OS/PFS of PASC patients.

In the univariate analysis of DFS time, the normal Ca199 level (p = 0.042), normal Ca125 level (p = 0.010) and no-smoking history (p = 0.019) were favorable prognostic factors. The p-values of the CEA level and positive symptoms were slightly higher than 0.05 (p = 0.085 and 0.164). Multivariate analysis showed that Ca199 <35 U/ml (HR = 0.464, 95% CI = 0.222–0.970, P = 0.041) and Ca125 <35 U/ml (HR = 0.441, 95% CI = 0.233–0.835, P = 0.012) were independent favorable prognostic factors. The results are displayed in **Table 3**. The Kaplan-Meier Curves were displayed in **Figure 1**.

**Table 3:** Multiple analysis of OS in PASC patients.

	Hazard ratio	95% CI	P value
Ca125 <35 U/ml	0.374	0.187–0.749	0.005
Ca199 <35 U/ml	0.387	0.168–0.890	0.025
Chemotherapy received	0.578	0.297–1.126	0.107
Smoking history	1.308	0.601–2.846	0.498
CEA <5 ng/ml	0.839	0.435–1.620	0.602

## Discussion

In this study, we identified and analyzed prognostic factors of patients with rPASC and found that serum levels of Ca199 and Ca125 were independent favorable prognostic factors. Patients with rPASC had a poor prognosis, and the 1-year, 3-year and 5-year survival rates were 40.9, 17.5, and 11.6%, respectively. Only four patients survived for more than 3 years, and two patients survived for more than 5 years. There have been no similar reports with over 50 cases to date. This study was also the first report to reveal the relationship between Ca125 and the survival of patients with PASC.

Pancreatic cancer is one of the most lethal malignancies, with an extremely poor prognosis and a 5-year survival rate of less than 5% (1, 7, 13). PASC is a rare subtype, accounting for only approximately 4% of pancreatic cancers (14). There are no typical symptoms in the early stages, making it difficult to diagnose. Some patients may experience digestive symptoms such as jaundice, abdominal pain, and nausea. Similar to PDAC, serum levels of tumor markers such as Ca199 are usually elevated in most patients (15, 16).

On enhanced CT scans, PASC usually appears similar to PDAC. On plain CT, PASC mostly manifests as low density (1, 16). The CT value of PASC in the arterial phase is lower than that of the normal pancreas. Unlike PDAC, necrosis and cystic changes can often be found on CT (17). Tumors of PASC patients are usually larger than those of PDAC patients, especially when the tumor is located in the neck or tail of the pancreas, due to a lack of typical symptoms (2, 6). Pathological findings provide the final basis for the diagnosis. The diagnostic criteria are that squamous cells make up at least 30% of all tumor cells (1).

In our study cohort, 54.5% (30/55) of patients had tumors located in the head of the pancreas, for whom pancreaticoduodenectomy was performed. Several studies have shown that a pancreatic head tumor is easier to detect because it can cause the occlusion of the pancreatic ducts and bile ducts, causing jaundice or acute pancreatitis (2, 18, 19). Other scholars believe that PASCs are the result of recurrent inflammatory stimulation (18). Ca199 and CEA were thought to be associated with PASC, but mechanisms underlying these connections remain controversial. The mean tumor size in this study was 4.5 ± 2.2 cm, and 56.4% (31/55) of patients had tumors larger than 4 cm, possibly because PASC always underwent cystic changes.

In our study, the median OS time and DFS time were 10 ± 2.1 and 4 ± 0.9 months, respectively, which were significantly worse than the long-term survival time of PDAC that was previously reported. There have been several previous articles on differences in OS and progression-free survival (PFS) between PDAC and PASC (2, 6, 7, 10). The histological difference between PASC and PDAC is the number of squamous cells in the pancreatic tumor tissue. There have been some reports in the past about the prognosis of PASC, especially in the Surveillance, Epidemiology, and End Results (SEER) database (11). However, in the present study, we reported our own experience and found that serum Ca199 and Ca125 levels were independent risk factors for OS and PFS in patients with rPASC. Chemotherapy use, smoking history, and the presence of abdominal symptoms were also associated with the prognosis of rPASC in univariate analyses but were excluded from multifactorial analyses. The chemotherapy regimens at our center are the AG or GS regimens, which are recommended by the guidelines (20). In Asian countries, the use of FOLFIRINOX is not popular because most Asians are intolerant of this regimen, and Asian patients seem to have a higher incidence of myelosuppression and impaired liver function. Therefore, AG or GS is preferred at our center. However, rPASC is not sensitive to chemotherapy and therefore has a worse prognosis than PDAC (2, 6, 7, 10). In a study of 203 patients with PASC in the SEER database, it was found that a combination with radiotherapy might be more effective in PASC patients (11). Immunotherapy or targeted therapy might be new options in the future. Due to the lack of a sufficient sample size, only retrospective studies can be conducted at this time, which may affect the results and conclusions.

Our study yielded some interesting results. From the results, tumor size and the presence or absence of lymph node metastases were not associated with patient prognosis, which was different from previous reports and other malignancies. The staging criteria for PDAC might not be applicable for patients with rPASC, as necrosis and cystic degeneration frequently occur in PASC. PASC tumors usually tended to be larger than PDAC tumors in previous reports (2, 6, 7, 10). Therefore, the T-staging system should be improved, and the N-staging system might also need to be modified, as there have been some reports in the past showing that lymph node metastases did not affect the prognosis of PASC, whereas PDAC did not. However, optimizing the staging system for PASC was not easy due to the lack of a sufficient sample size.

### Limitations

Since the study was retrospective and the sample size was not large enough, the results might be biased. PASC is currently underreported, so the data available are very limited, and larger sample sizes would need to be studied in the future to obtain more accurate results.

## Conclusion

Patients with PASC had poor survival. The 5-year survival was only 11.6%. Normal serum levels of Ca199 and Ca125 were independent favorable prognostic factors. However, the results were limited because of the lack of enough patients. Larger sample sizes and longer follow-up times would be helpful to obtain more accurate results.

## Data Availability Statement

The original contributions presented in the study are included in the article/supplementary material. Further inquiries can be directed to the corresponding authors.

## Ethics Statement

The studies involving human participants were reviewed and approved by The institutional review board of Shanghai Ruijin Hospital. The patients/participants provided their written informed consent to participate in this study.

## Author Contributions

The conception and design of the study: YS, BS. Acquisition of data: XW, JX. Analysis and interpretation of data: JJ, CP. Drafting the article: YS, WW, XD. Final approval of the version to be submitted: HC, XD, BS. All the work was accomplished by co-authors and there is no conflict of interest to disclose. All authors contributed to the article and approved the submitted version.

## Funding

The study was funded by Shanghai Sailing Program directly to YS (Grant number: 20YF1427800) and National Natural Science Foundation of China (NSFC) directly to BS (Serial number: 81871906).

## Conflict of Interest

The authors declare that the research was conducted in the absence of any commercial or financial relationships that could be construed as a potential conflict of interest.
